# Non-invasive MR imaging of inflammation in a patient with both asymptomatic carotid atheroma and an abdominal aortic aneurysm: a case report

**DOI:** 10.1186/1750-1164-1-4

**Published:** 2007-02-21

**Authors:** Simon PS Howarth, Tjun Y Tang, Martin J Graves, Jean-Marie U-King-Im, Zhi-Yong Li, Stewart R Walsh, Michael E Gaunt, Jonathan H Gillard

**Affiliations:** 1 University Department of Radiology, Cambridge University Hospitals NHS Foundation Trust, UK; 2 Academic Department of Neurosurgery, Cambridge University Hospitals NHS Foundation Trust, UK; 3 Cambridge Vascular Unit, Cambridge University Hospitals NHS Foundation Trust, UK

## Abstract

Inflammation is a recognized risk factor for the vulnerable atherosclerotic plaque.

USPIO-enhanced MRI imaging is a promising non-invasive method to identify high-risk atheromatous plaque inflammation in vivo in humans, in which areas of focal signal loss on MR images have been shown to correspond to the location of activated macrophages, typically at the shoulder regions of the plaque. This is the first report in humans describing simultaneous USPIO uptake within atheroma in two different arterial territories and again emphasises that atherosclerosis is a truly systemic disease. With further work, USPIO-enhanced MR imaging may be useful in identifying inflamed vulnerable atheromatous plaques in vivo, so refining patient selection for intervention and allowing appropriate early aggressive pharmacotherapy to prevent plaque rupture.

## Background

It is accepted that vulnerable atheromatous plaque has a thin fibrous cap and large lipid core with associated inflammation [1]. This inflammation can be detected on Magnetic Resonance (MR) imaging using Ultra Small Super-Paramagnetic Iron Oxide (USPIO) particles as a contrast medium (Sinerem™). This has been validated against the histological gold standard in previous work [2]. Ex-vivo imaging of atheroma allows an extended imaging-time thereby permitting greater signal-to-noise and improving plaque characterisation. This has been shown to correlate well with in-vivo imaging.

## Case Presentation

A 72-year old male was referred to the vascular outpatient clinic with an asymptomatic right carotid bruit, for work up before planned coronary artery bypass grafting. His duplex ultrasound showed a 70–80% stenosis of the right internal carotid artery. He was found also to have an incidental 5.6 cm infra-renal abdominal aortic aneurysm (AAA). He underwent multi-sequence MR imaging pre- and 36 hours post USPIO infusion. Multi spectral imaging was acquired at 1.5 Tesla using a whole body system (GE Medical Systems, Milwaukee) and a custom designed 4-channel phased array neck coil (Flick Engineering Solutions BV) along with a standard body coil to improve signal to noise ratio. The patient subsequently (24 hours) underwent a right carotid endarterectomy and was discharged home after an uncomplicated hospital stay of three days.

**Figure 1:**
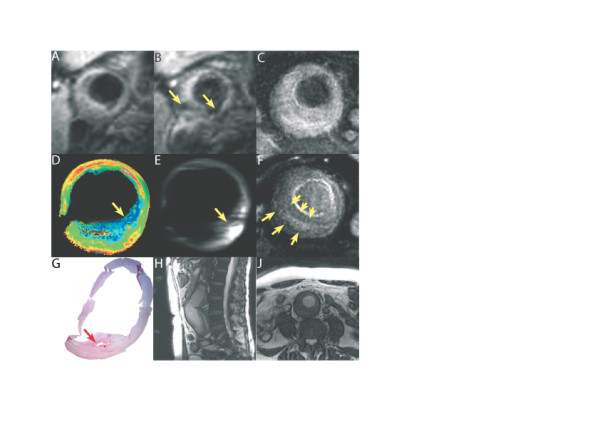
T_2_* weighted spiral imaging of the right common carotid artery *in-vivo*, pre (A) and 36 hours post USPIO infusion (B) showing signal loss in areas of USPIO uptake (yellow arrows). (The 2D T_2_* weighted spiral acquisition used a spectral-spatial excitation pulse, with a TE of 5.6 ms. The multi-shot spiral sequence involved the acquisition of 22 spiral interleafs each of 4096 data points resulting in an effective in-plane pixel size of 0.42 × 0.42 mm, two signal averages were performed and a quadruple inversion preparation was utilised to null the signal from blood pre – and post USPIO. Slices were acquired sequentially with a 3 mm thickness and no inter-slice gap.). Pre (C) and 36 hours post USPIO infusion (F) T_2_* weighted spiral imaging *in-vivo *revealing signal drop in the wall of the aneurysm post-USPIO (yellow arrows) likely corresponding to regions with a high inflammatory burden. Corresponding *ex-vivo *imaging in a dedicated micro-coil with T_2 _map (D) showing regions with very short T_2 _species (yellow arrow) corresponding with area of USPIO uptake. Ex-vivo inversion recovery on-resonance water suppression (IRON) imaging [9] (E) with off-resonant spins showing positive contrast due to dephasing of spins adjacent to USPIO uptake. H&E section (x40) co-registered with ex-vivo imaging (using distance from the bifurcation). Area of intraplaque haemorrhage within a small necrotic lipid core can be seen (red arrow), adjacent to the USPIO uptake seen in the ex-vivo imaging. Structural MR Imaging in the same patient reveals anatomy of the co-existing abdominal aortic aneurysm (H&J).

The ex-vivo specimen was kept fresh and immersed in an MR inert fluid (Fomblin, Performance Fluids Ltd, UK) and imaged in a dedicated single channel micro-coil 4 hours after surgery. Following this, the specimen was washed, fixed in formalin and embedded in paraffin wax. The paraffin block was divided into 3 mm sections and thin sections were cut from each block. These sections were stained using various techniques including haematoxylin and eosin (H&E) and elastin Van Giesen (EVG). This histology was co-registered with the ex-vivo and in-vivo MR using the bifurcation as a point of reference. There were no problems with the co-registration of the in-vivo images with the ex-vivo images or the histology (see Figure 1).

An elective endovascular repair of his AAA has been scheduled.

## Conclusion

Treatment decisions for surgical intervention in patients with asymptomatic carotid atheroma remain controversial. Conventional clinical risk assessment of carotid atheroma is based currently on luminal stenosis alone [3]. Although important, methods used to measure luminal stenosis such as conventional x-ray angiography and CT angiography do not adequately reflect disease severity in carotid atherosclerosis. They do not permit assessment of the morphology and inflammatory infiltrate of the lesion, which are all recognised risk factors for the vulnerable carotid atheromatous plaque [4]. Furthermore, the process of expansive arterial remodelling [5] may produce normal luminal measurements despite a large atheromatous plaque burden in a particular patient and therefore underestimate their risk of stroke.

USPIO-enhanced MRI imaging is a promising non-invasive method to identify high-risk atheromatous plaque inflammation in vivo in humans, in which areas of focal signal loss on MR images have been shown to correspond to accumulation of iron particles in ex vivo specimens [2,6]. USPIO is thought to accumulate predominantly in activated macrophages either at the shoulders or in the necrotic lipid core of ruptured and rupture-prone human atherosclerotic lesions and is considered to be a marker of the degree of inflammation within the plaque [7,8].

This is to the authors' knowledge the first report in humans describing simultaneous USPIO uptake within atheroma in two different arterial territories and again emphasises that atherosclerosis is a truly systemic disease.

With further work, USPIO-enhanced MR imaging may be useful in identifying inflamed vulnerable atheromatous plaques in vivo, so refining patient selection for intervention and allowing appropriate early aggressive pharmacotherapy to prevent plaque rupture. It may also prove a useful adjunct in the identification of inflammation within other vascular beds such as in the detection of inflammatory abdominal aortic aneurysms, which may contribute to surgical decision making in the future.

## Competing interests

JHG is a consultant to GlaxoSmithKline

## Authors' contributions

SH jointly had the original idea, drafted the manuscript and designed the Figure. TT jointly had the original idea, was involved in the drafting and editing of the manuscript and Figure. MJG carried out the MR sequence development and helped in drafting the manuscript. JMUKI participated in the design of the study and edited the manuscript. ZYL helped in editing the manuscript and creating the Figure. SRW was involved with manuscript editing, patient care and helped perform the operation. MEG edited the manuscript, was the clinical lead in the patient's care and was the primary surgeon in performing the carotid endarterectomy. JHG helped conceive the study, was involved in the radiology reporting of the patient's MR images and edited the manuscript. All authors have read and approved the final manuscript.

## Sources of funding

GlaxoSmithKline and The Stroke Association
